# Morphogenesis of pteraspid heterostracan oral plates and the evolutionary origin of teeth

**DOI:** 10.1098/rsos.240836

**Published:** 2024-12-18

**Authors:** Madleen Grohganz, Zerina Johanson, Joseph N. Keating, Philip C. J. Donoghue

**Affiliations:** ^1^ Palaeobiology Research Group, School of Earth Sciences, University of Bristol, Life Sciences Building, Tyndall Avenue, Bristol BS8 1TQ, UK; ^2^ Natural History Museum, Cromwell Road, London SW7 5BD, UK

**Keywords:** early vertebrates, heterostracans, evolution, teeth, histology, morphogenesis

## Abstract

Teeth are a key vertebrate innovation; their evolution is generally associated with the origin of jawed vertebrates. However, tooth-like structures already occur in jawless stem-gnathostomes; heterostracans bear denticles and morphologically distinct tubercles on their oral plates. We analysed the histology of the heterostracan denticles and plates to elucidate their morphogenesis and test their homology to the gnathostome oral skeleton. We identified a general model of growth for heterostracan oral plates that exhibit proximal episodic addition of tubercle rows. The distal hook exhibits truncated lamellae compatible with resorption, but we observe growth layers to be continuous between denticles. The denticles show no evidence of patterns of apposition or replacement indicating tooth homology. The oral plates and dermal skeleton share the same histological layers. The denticles grew in a manner comparable to the oral plate tubercles and the rest of the dermal skeleton. Our test of phylogenetic congruence reveals that the distribution of internal odontodes is discontinuous, indicating that the capacity to form internal odontodes evolved independently several times among stem-gnathostomes. Our results support the ‘outside-in’ hypothesis and the origin of teeth through the spread of odontogenic competence from extra-oral to oral epithelia and the subsequent co-option to a tooth function in gnathostomes.

## 1. Introduction

Teeth are a key innovation that underpinned the evolutionary and ecological diversification of jawed vertebrates [1], as well as a model system for organ development [2]. The epithelial–mesenchymal interactions governing tooth formation are also active during the development of other organs like hair follicles and mammary glands [3–7]. Minimally, teeth are composed of dentine, and they may be covered with a hard tissue (e.g. enamel and enameloid) and are attached to the jawbone by bone or ligament, with functional teeth being replaced via loss or sequential addition (in crown-gnathostomes) [8]. Besides extant jawed vertebrates, the extinct placoderm groups Arthrodira (*Compagopiscis*) and Acanthothoraci (*Romundina*) also possessed teeth (primitively, at least) [9,10], and so we can infer teeth to have already been present in the last common ancestor of all known jawed vertebrates. Like jaws, teeth must have a deeper evolutionary history, but the evolutionary relationship of teeth to tooth-like structures in jawless vertebrates is unclear [4,11,12].

Two main hypotheses have been proposed to explain the evolutionary origins of teeth, the ‘outside-in’ (see review in Donoghue & Rücklin [11]) and ‘inside-out’ hypotheses [13–17], which differ in terms of the origins of tooth precursors, from external dermal scales versus internal denticles that have a distinct evolutionary origin, respectively. Here we aim to evaluate these hypotheses through analysis of the development of the oral plates associated with one of the earliest lineages of skeletonizing vertebrates, the heterostracans.

The inside-out hypothesis was evidenced by the interpretation of the tooth-like conodont ‘elements’ as composed of an enamel-like cap and a dentine-like base, which appeared prior to the dermal skeleton [14–17]. However, the enamel-like cap is specific to euconodonts, a derived group of conodonts, and thus conodont elements are not homologous with teeth [11,18].

The presence of oral and pharyngeal denticles in the extinct jawless thelodonts [19] was also presented in support of the inside-out hypothesis, interpreted as tooth-like and exhibiting sequential replacement [14–17]. However, thelodonts are phylogenetically remote from dentate jawed vertebrates, and phylogenetic intermediates and sister lineages have been interpreted as edentate [11,14,16,17,20]. As such, the proposition of homology between the teeth of jawed vertebrates and the oral and pharyngeal denticles of thelodonts fails a test of phylogenetic congruence [11,20,21]. However, many of the phylogenetic intermediates and sister lineages of jawed vertebrates and thelodonts possess denticles associated with their oral and pharyngeal openings, including the median dorsal field of osteostracans [22], the large dorsal nasohypophyseal opening of galeaspids [19] and the oral plates of pteraspid heterostracans; heterostracans are among the earliest skeletonizing vertebrates [23,24] and, therefore, among the earliest lineages in which a distinction between oral and external odontodes—evolutionary precursors of teeth and dermal scales could be drawn.

**Figure 1.**
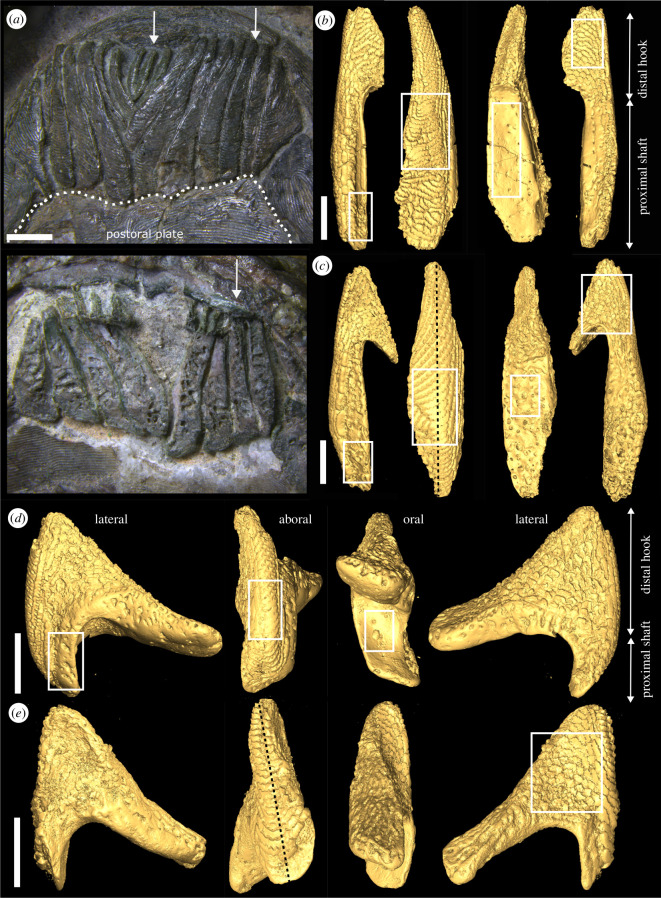
(*a*) Articulated, V-shaped oral plate apparatus of *Protopteraspis vogti* [30]: top, aboral view; bottom, oral view; arrows mark individual median (left) and lateral (right) oral plates. (*b*) Three-dimensional surface models of *Loricopteraspis dairydinglensis* lateral oral plate (specimen NHMUK PV P 43713): from left to right, lateral view with rectangle indicating vascular spaces opening up to the lateral surface, aboral view with rectangle indicating abraded tubercles, oral view with rectangle indicating vascular spaces opening up to the oral surface, lateral view with rectangle indicating position of close-up in figure 2*b*. (*c*) Three-dimensional surface models of *L. dairydinglensis* lateral oral plate (specimen NHMUK PV P 43711): from left to right, lateral view, with rectangle indicating vascular spaces opening up to the lateral surface, aboral view with rectangle indicating abraded tubercles and line representing position of the tomographic section in figure 2*j*, oral view with rectangle indicating vascular spaces opening up to the oral surface, lateral view with rectangle indicating position of close-up in figure 2*a*. (*d*) Three-dimensional surface models of *L. dairydinglensis* median oral plate (specimen NHMUK PV P 43709): from left to right, lateral view with rectangle indicating vascular spaces opening up to the lateral surface, aboral view with rectangle indicating abraded tubercles, oral view with rectangle indicating vascular spaces opening up to the oral surface, lateral view. (*e*) Three-dimensional surface models of *Pteraspis* sp. median oral plate (specimen NHMUK PV P 76697): from left to right, lateral view, aboral view with line representing position of tomographic section in figure 2*l*, oral view, lateral view with rectangle indicating position of close-up in figure 2*c*. Scale bars represent: 2 mm (*a*); 1000 μm (*b–e*).

**Figure 2.**
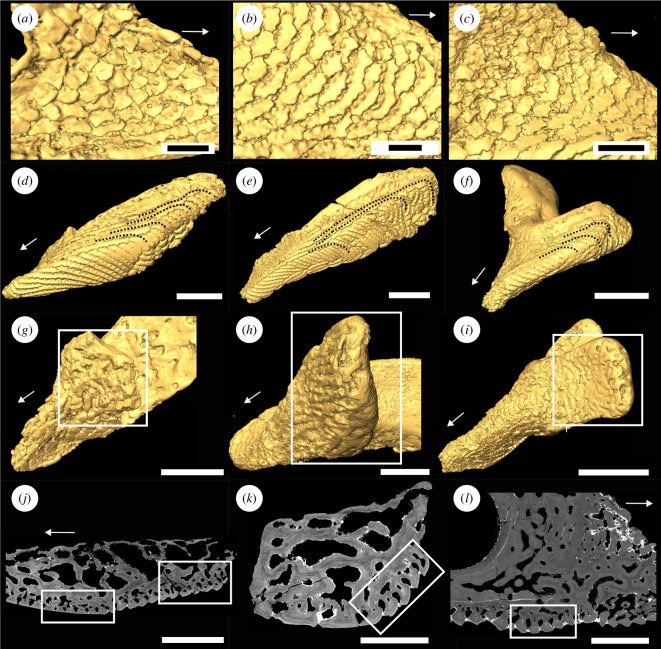
(*a–c*) Close-ups of denticles on the lateral hook surfaces of oral plate specimens shown in figure 1: (*a*) lateral oral plate specimen NHMUK PV P 43711; (*b*) lateral oral plate specimen NHMUK PV P 43713; (*c*) median oral plate specimen NHMUK PV P 76697; (*d–f*) aboral-lateral view showing tubercles on the lateral surface bending to the aboral side and connecting to the aboral tubercles (indicated by dotted lines): (*d*) lateral oral plate specimen NHMUK PV P 43711; (*e*) lateral oral plate specimen NHMUK PV P 43713; (*f*) median oral plate specimen NHMUK PV P 43709; (*g–i*) close-up of oral hook surface with abrasion on distal tip (rectangle): (*g*) lateral oral plate specimen NHMUK PV P 43711; (*h*) median oral plate specimen NHMUK PV P 76697; (*i*) median oral plate specimen NHMUK PV P 43709; (*j–k*) tomographic sections through the shaft of lateral oral plate specimen NHMUK PV P 43711: (*j*) showing abraded tubercles on the aboral surface (left rectangle) and less infilled tubercles at the proximal shaft end (right rectangle); (*k*) showing infill of tubercles on the lateral surface (rectangle) with aboral tubercles to the bottom and oral tubercles to the top of the image; (*l*) tomographic section through median oral plate specimen NHMUK PV P 76697 showing non-abraded tubercles on the aboral surface (rectangle). Arrows indicate distal direction, and scale bars represent: 300 μm (*a,c*); 200 μm (*b*); 1000 μm (*d–g,i*); 500 μm (*h,k–l*); 900 μm (*j*).

Here we investigate the structure and morphogenesis of pteraspid heterostracan oral plates and their associated denticles to explore their possible homology with the teeth of jawed vertebrates. The oral plates are best known in pteraspids [23,25–28], which possess a V-shaped apparatus of small, narrow, rod-like oral plates [29] (figure 1*a*). The lateral sides of the distal hook-like part of these oral plates are covered with (patterned) rows of triangular or maple-leaf-shaped, sharply pointed denticles, which are facing rostrally (out of the oral cavity) [31] (see also figure 2*a*–*c*). Some authors have argued that the oral plates were used in active, predatory feeding (see [27] for a recent summary), and as the oral plates evidence denticles associated with the mouth opening, they are potentially compatible with the outside-in hypothesis.

### 1.1. Hypotheses of homology and evolution of teeth

While there may be some disagreement over the evolutionary origin of teeth, researchers are united in recognizing oral teeth and scales, as well as dentine-bearing dermal scales tubercles and denticles as classes of odontode. The odontode concept has itself evolved over time [4], but its current usage follows Reif [8], recognizing structures that are the product of an undivided dental papilla bounded by an epithelial dental organ, composed of dentine, usually with an enamel or enameloid cap and bone of attachment [8,32,33]. Odontodes can be internal, located inside the oropharyngeal cavity (oral/pharyngeal/nasal denticles, teeth) or external, located outside the oropharyngeal cavity (dermal denticles (scales), tubercles). Thus, different classes of odontodes can be recognized; here we distinguish teeth, denticles and tubercles. Teeth are internal odontodes, attached to a jaw by bone or ligament, and may be replaced by loss or sequential addition. Denticles are odontodes with an asymmetrical and pointed morphology that can be located externally as dermal denticles (scales) or internally as oral, pharyngeal or nasal denticles. Tubercles are odontodes with a rounded morphology that can also occur in ridges but occur principally as part of the dermal skeleton.

The long-standing hypothesis for the origin of teeth has been coined the ‘outside-in’ hypothesis. It proposes that teeth evolved in conjunction with the origin of jaws. They developed from tooth-like dermal denticles (external odontodes), extending their distribution from the external dermis to the internal oral cavity and adapting to a tooth function. This theory is based on similarities in the structure and development of dermal denticles with teeth [34] and their shared development from a homologous unit termed the odontode [4,8,32,33]. Teeth and placoid scales in extant chondrichthyans are often cited as a textbook example for this homology [12,35,36]. Both external dermal and oral odontodes seem to develop as comparable developmental modules, only modulated by heterotopy (i.e. a change in the initiation site of a common gene regulatory network) [37]. Huysseune *et al*. [38] proposed a revised ‘outside-in’ hypothesis emphasizing the homology of dermal denticles and teeth and the ectodermal origin of dental epithelium. They propose that teeth evolved prior to jaws (like in the inside-out hypothesis), but from ectoderm invading the oropharyngeal cavity through mouth and gill slits and transferring its odontogenic competence to the endoderm. Later in evolutionary history the ectoderm invasion would have become more spatially restricted, and the endoderm would have been co-opted to take up the odontogenic fate of the ectoderm [11,15,39,40].

The ‘inside-out’ hypothesis proposes that teeth evolved independently from and prior to jaws and have an evolutionary history preceding the dermal skeleton [13–17]. Oral teeth are hypothesized to have been co-opted from endodermal pharyngeal denticles (internal odontodes). Extinct jawless vertebrates possessed diverse feeding structures, and it has been argued that the teeth of gnathostomes may have evolved from these specialized tooth-like structures in the oropharyngeal cavity of jawless vertebrates independent of the dermal skeleton. Key evidence for the ‘inside-out’ hypothesis included the claims that endoderm is required for tooth development [16,41,42], the interpretation of conodont elements as the first oropharyngeal denticles prior to the origin of jaws [16] and the discovery of patterned tooth whorls in thelodonts [19], suggesting the presence of a dental lamina [8]. All of these arguments have been refuted [11,18,38,40,43], undermining the ‘inside-out’ hypothesis.

In attempting to test among these hypotheses, it is pertinent to consider the basis under which tooth homology can be established. What is the difference between a tooth and a merely tooth-like structure? The following four main criteria have been employed previously to identify teeth and tooth homologues:

- Topology: teeth are located in the oropharynx and develop from oral and/or pharyngeal epithelium. An association with the jaw is not necessary, as teeth and jaws have been shown to be entirely independent developmental modules [44], and so there should be no expectation that teeth and jaws originated together. This fact allows for the possibility that teeth evolved before jaws in the oropharynx, which is a core component of the ‘inside-out’ hypothesis. However, not all tooth-like structures located in the oropharynx qualify as teeth. For example, the post-branchial lamina of a range of placoderms carries patterned arrays of denticles [45] that are not teeth but focal developments of continuous sheets of spongy bone added episodically [10]. Therefore, oropharyngeal topology is necessary, but on its own not a sufficient criterion for establishing tooth homology.
- Structure/composition: minimally teeth are composed of dentine with a pulp cavity, which is successively infilled. They may be covered with hard tissue (e.g. enamel and enameloid), but some teeth also lack a cover of hypermineralized tissue like in the teeth of the arthrodiran placoderm *Compagopiscis* [10]. This reduced structure is not restricted to teeth but characterizes all odontodes, i.e. also the ones in the dermal skeleton, e.g. of heterostracans [23]. Therefore, structure is again a necessary but on its own not a sufficient criterion for establishing tooth homology.
- Function: teeth perform an active mechanical function related to feeding. This has been invoked in arguments over the homology of teeth in assessing isolated skeletal elements from the enigmatic *Lophosteus* and *Andreolepis* [46–48]. However, teeth do not always perform a mechanical function, e.g. crabeater seals possess finely divided, lobed teeth, which are used for filter feeding on krill instead of an active mechanical function [49]. On the other hand, structures like the bony jaws projections of the 'false-toothed' birds, the pelagornithids [50], may have performed a tooth function, even though they are clearly not teeth because they do not develop from the oropharynx and lack odontode structure, which, minimally, should be composed of dentine. Therefore, function is neither a necessary nor a sufficient criterion on which to identify tooth homology.
- Development: a commonly reported feature of teeth is that they develop ahead of functional need and from within a protected location known as the dental lamina (where the epithelial and mesenchymal tissues are located) from which replacement teeth emerge [8]. This expectation is strongly biased by the pattern of tooth replacement in sharks, which has long been assumed, incorrectly, to be the ancestral condition for tooth development and replacement [51]. This ignores the fact that replacement teeth often develop from a discontinuous and transient dental lamina or from a superficial position in the absence of a dental lamina [40]. Tooth replacement also occurs without tooth shedding, with the apposition of new teeth onto the margins of their precursors [10,51,52]. In addition, teeth develop as discrete morphogenetic modules, as do some dermal tubercles, but dermal tubercles often develop in concert, with evidence of continuous growth between adjacent odontodes. Development presents the strongest criterion for tooth homology; teeth are characterized by patterns of replacement (either via tooth loss or sequential apposition) and lack of evidence of continuous growth between adjacent odontodes. However, while these developmental characteristics are a necessary condition for tooth homology, they are not sufficient on their own, since such conditions can also be met in the dermal skeleton.

Our review indicates that function cannot be used reliably as a criterion for tooth homology, as not all teeth perform a tooth function and not all structures performing a tooth function are teeth. While the remaining criteria are individually necessary but not sufficient, in combination, structure/composition, topology and development are sufficient to establish a hypothesis of primary homology. However, they remain insufficient to establish homology since the concept of homology encompasses historicity. Patterson [53] and de Pinna [54] have both argued persuasively that criteria such as those above simply support a conjecture of homology (or primary homology) and that a hypothesis of homology should equate with a synapomorphy, making homology the property of a monophyletic group. This links the concept of homology to phylogeny and enables us to test homology in a systematic context.

In this light, we assess the homology of gnathostome teeth and heterostracan oral plate denticles on the basis of topological, structural/compositional and inferred developmental similarity, in comparison to external dermal denticles. Primary conjectures of homology will be evaluated post hoc for phylogenetic congruence. Phylogenetic congruence would be compatible with the ‘inside-out’ hypothesis, while repeated evolution of such structures, failing a test of phylogenetic congruence, would be compatible with the ‘outside-in’ hypothesis of tooth evolution.

## 2. Material and methods

To characterize the structure and development of the pteraspid oral plates and dermal skeleton, we used synchrotron radiation X-ray tomographic microscopy (srXTM; [55]); (see electronic supplementary material, S1). Based on srXTM scans, we created three-dimensional tomographic models and analysed virtual sections taken through the models to characterize histology and morphogenesis of the oral plates and denticles and overall oral plate growth for comparison to the dermal skeleton. Tomographic models are based on isolated oral plates (specimens NHMUK PV P 43713, NHMUK PV P 43711, NHMUK PV P 43709 and NHMUK PV P 43710) and dermal skeleton fragments (NHMUK PV P 77921 and NHMUK PV P 77922) of *Loricopteraspis dairydinglensis*, collected from the Lower Devonian, Ditton Series, Dairy Dingle locality near Neenton, Shropshire, UK [56], and isolated oral plates (specimens NHMUK PV P 76697, NHMUK PV P 76943 and NHMUK PV P 76944) of *Pteraspis* sp., collected from the Lower Devonian, Ditton Series, New Inn 2 locality, near Upper Hayton, Shropshire, UK [56]. All examined materials are housed in the Natural History Museum, London (NHMUK).

We focus on these taxa because isolated oral plates from heterostracans are generally rare, and oral plates from these taxa have been the focus of previous analyses of oral plate function in heterostracans (e.g. [27,31]). Furthermore, their oral plates are considered a representative feature of pteraspidiform heterostracans, rendering conclusions derived from *L. dairydinglensis* and *Pteraspis* generally relevant to the group as a whole [57]. We adopt the following terminology to describe the nature and arrangement of the oral plates [58]: we use lateral/median to describe the position of the oral plates in the apparatus; oral/aboral to describe the orientation of plate surfaces relative to the oral cavity, the oral surface with open vascular canals facing the oral cavity and the aboral surface with tubercles facing away from the oral cavity; proximal/distal to refer to the position on the oral plates relative to their junction with the postoral plate (see figure 1*a*), proximal being closer to this junction and distal further.

## 3. Results

### 3.1. Oral plate morphology

The oral plates have different morphologies depending on their position in the apparatus: the lateral plates at the margins of the oral plate apparatus (see figure 1*a*) are more asymmetrical with a longer proximal shaft compared to the more distal hook (figure 1*b*,*c*). Most proximally, the lateral plates articulate directly with the postoral plate (figure 1*a*). The median plates sit in the middle of the apparatus (see figure 1*a*) and are more symmetrical; the distal hook and the proximal shaft are relatively similar in length (figure 1*d*,*e*). The median plates do not articulate with the postoral plate (see figure 1*a*), but abut the lateral surfaces of the neighbouring oral plate (see [58]).

In both the lateral and median plates, the oral plate surfaces are covered with two different types of structures, denticles and tubercles. In accordance with Purnell [31], we observe that the denticles can be found on the lateral surfaces of the distal hook and are rostrally facing (out of the oral cavity; figure 1*b*–*e* lateral views and figure 2*a*–*c*). Contrasting Purnell [31], we find a range of denticle morphologies from sharp, pointed, triangular or maple-leaf-shaped (as described by Purnell [31, fig. 2*c*,*d*]) to abraded (figure 2*a*–c; see also [27]).

The tubercles occur as interlocking ridges on the aboral side of the oral plate and on the lateral surfaces of the oral plate shaft (figure 1*b*–*e* aboral views and figure 2*d*–*f*). The tubercle rows on the lateral surface bend towards the aboral and are connected to the tubercle rows here (figure 2*d*–*f*, dotted lines). In the lateral oral plates examined, the tubercles are abraded around the mid-aboral surface (as observed by Purnell [31]). This is evident from the surface models (figure 1*b*–*c* aboral views, rectangles) as well as a tomographic section where the top of the tubercles has been removed (figure 2*j*, left rectangle). In the median oral plates, only one specimen shows abrasion of the tubercle ridges on the aboral surface (figure 1*d* aboral view, rectangle). No abrasion is visible on the aboral surface of the other specimen (figure 2*l*, rectangle).

The upper half of the lateral surfaces of the shaft as well as the oral surface of the shaft and the proximally facing side of the distal hook are unornamented and not covered in tubercles or denticles, instead vascular spaces open at the surface of the oral plate (figure 1*b*–*d* oral views, rectangles). In all specimens (except for NHMUK PV P 43713, where the hook is missing), on the lateral as well as median oral plates, the distal tip of the hook is abraded (figure 2*g*–*i*, rectangles).

### 3.2. Oral plate histology

**Figure 3.**
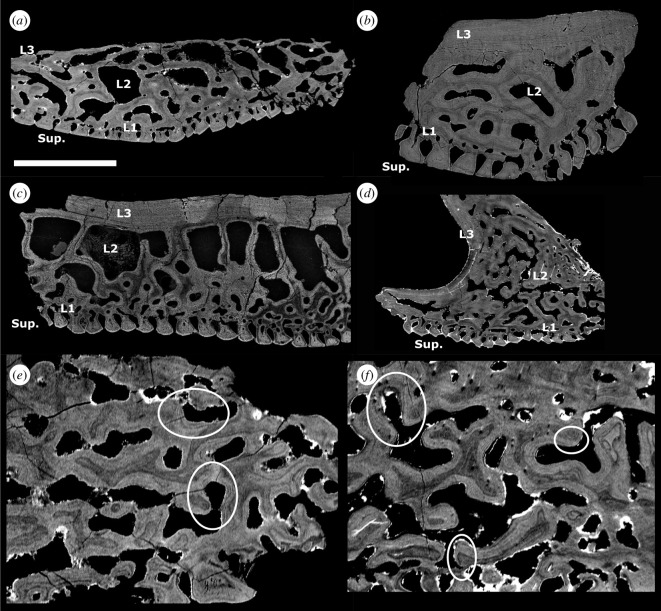
(*a*) Tomographic longitudinal section through the oral plate shaft of *L. dairydinglensis* lateral oral plate (specimen NHMUK PV P 43711); (*b*) tomographic cross-section through the oral plate shaft of *Pteraspis* sp. lateral oral plate (specimen NHMUK PV P 76943); (*c*) scanning electron microscopy BSE of section through *L. dairydinglensis* cephalothoracic shield (NHMUK P.73622) (based on [23]); (*d*) tomographic section of *Pteraspis* sp. median oral plate (specimen NHMUK PV P 76697); (*e*) tomographic section through *L. dairydinglensis* lateral oral plate (specimen NHMUK PV P 43711) showing resorption of the internal microstructure of the hook (circles); (*f*) tomographic sections of *Pteraspis* sp. median oral plate (specimen NHMUK PV P 76697) showing resorption of the internal microstructure of the hook (circles). Scale bar represents: 1000 μm (*a,b,d*); 802 μm (*c*); 240 μm (*e*); 212 μm (*f*).

The heterostracan oral plates are generally four-layered and comprise a superficial layer composed of tubercles and denticles, a compact layer of canals, a cancellous layer and a plywood-like layer (see figure 3*a*–*b,d*, Sup., L1−3, respectively).

#### 3.2.1. Superficial layer

This layer comprises tubercles and denticles, approximately 60 μm (denticles) to 120 μm (tubercles) thick. On the aboral and lateral surface of the oral plate shaft, the superficial layer consists of tubercle ridges. The outer margins of the superficial tubercle ridges are serrated so that they interlock and completely enclose the flask-shaped grooves separating them (figure 1*b*–*e* aboral views; figure 2*d*–*f, j,l*; figure 3*a*–*b,d*, Sup.). The oral plate tubercles are covered with a cap of enameloid approximately 5–10 μm thick (figure 3*a*–*b,d*). Below the enameloid cap, the tubercles consist of dentine growing centripetally towards pulp canals. In the lateral oral plate, tubercles comprising the tubercle rows on the lateral shaft surfaces show different degrees of dentine infilling; tubercles in aboral rows are more infilled than tubercles in oral rows (figure 2*k*, rectangle). The tubercle ridges on the aboral surface show a similar pattern, with the tubercles in proximal rows being less infilled than the ones in distal rows (figure 2*k*, rectangle).

**Figure 4.**
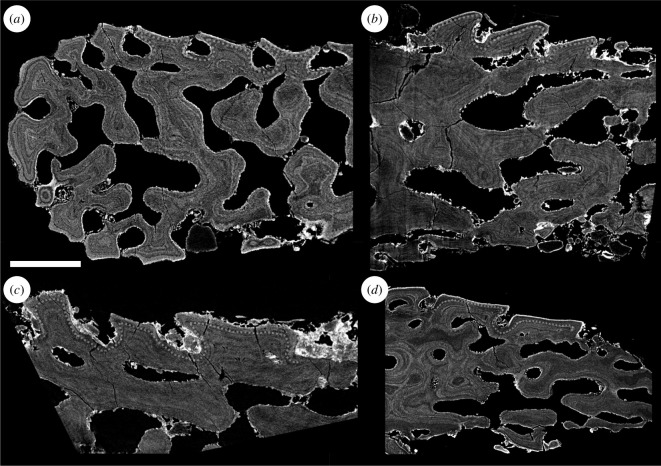
Tomographic sections through high-resolution scans of denticles on the lateral surfaces of the distal hook in different oral plates (perpendicular to rows of denticles): (*a,b*) denticles on lateral oral plate NHMUK PV P 76944; (*c*) denticles on lateral oral plate NHMUK PV P 76943; (*d*) denticles on lateral oral plate NHMUK P 43710. Dotted lines indicate continuous growth lines between individual denticles. Scale bar represents: 100 μm (*a–d*).

On the lateral surfaces of the distal hook, the superficial layer consists of denticles (figure 1*b*–*e* lateral views; figure 2*a*–*c*). But instead of regular symmetrical interlocking ridges they form rows of asymmetrical rostrally facing structures with open spaces between the individual denticles (see also two-dimensional denticle models in [27]). The denticles show the same histology as the tubercles with centripetally growing layers of dentine covered by an enameloid cap (figure 4). The dentine layers inside the denticles are continuous across denticle rows (figure 4, dotted lines, see also electronic supplementary material, S3). There is no evidence for replacement or apposition of denticles.

#### 3.2.2. Compact layer of canals

This layer is discontinuous across the oral plate, up to approximately 50–80 μm in thickness, and comprises vascular canals (figure 3*a*–*b*,*d*, L1).

#### 3.2.3. Cancellous layer

This layer is approximately 500–800 μm thick. In the proximal oral plate shaft, this layer consists of big, open cancellar spaces separated by walls (figure 3*a*–*b*,*d*, L2). In the distal oral plate hook, this layer has a different architecture and comprises densely packed cancellar spaces (figure 3*d*). The lamellar walls of the dense cancellar spaces in the oral plate hook are often truncated, which indicates resorption (figure 3*e*,*f*; circles, see also electronic supplementary material, S2).

#### 3.2.4. Plywood-like layer

This layer is approximately 50–100 μm thick. It overlies the cancellous layer and consists of plywood-like lamellar layers (figure 3*a*,*b*,*d*, L3). Cross-sections of the shaft show successive lamellar layers being added towards the oral surface in this layer (figure 3*a*,b). A similar successive apposition of growth layers can be observed in the plywood-like layer on the proximally facing side of the hook (figure 3*d*).

### 3.3. Dermal skeleton histology

**Figure 5.**
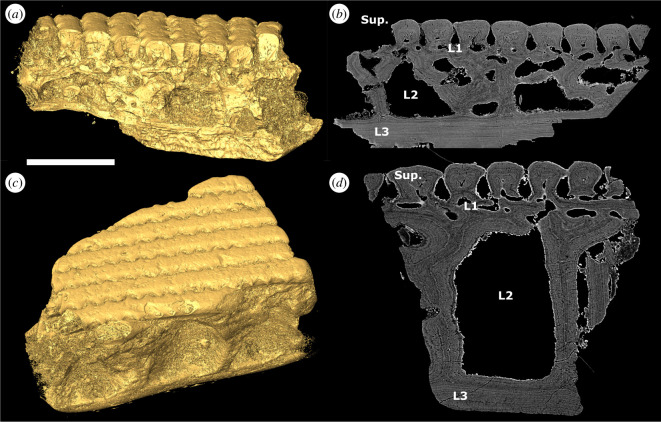
Three-dimensional surface models and tomographic sections of *L. dairydinglensis* dermal skeleton fragments: (*a*) surface model of dermal skeleton fragment NHMUK PV P 77921; (*b*) tomographic section through dermal skeleton fragment NHMUK PV P 77921; (*c*) surface model of dermal skeleton fragment NHMUK PV P 77922; (*d*) tomographic section through dermal skeleton fragment NHMUK PV P 77922. Scale bar represents: 500 μm (*a–c*); 300 μm (*d*).

The heterostracan dermal skeleton is generally four-layered and is composed of a superficial layer consisting of tubercles, a compact layer of canals, a cancellous layer and a plywood-like layer (figure 5, Sup., L1−3, respectively).

#### 3.3.1. Superficial layer

This layer is approximately 120–150 μm thick and comprised of only tubercles and no denticles. Similar to the oral plates, the outer margins of the superficial tubercle ridges are serrated and interlocking, completely enclosing the flask-shaped grooves separating them (figure 5). They show the same histology as the oral plate denticles and tubercles, a cap of enameloid approximately 5 μm thick over dentine growing centripetally towards pulp canals, from which small canals (canaliculi) radiate.

#### 3.3.2. Compact layer of canals

This layer is approximately 50 μm thick, similar to the oral plates, and comprises vascular canals (figure 5).

#### 3.3.3. Cancellous layer

This layer is approximately 350–500 μm thick, thinner than in the oral plates. It consists of big, open cancellar spaces (figure 5). In contrast to the cancellous layer in the oral plate hook, the growth layers of this layer are not truncated by vascular spaces and no evidence for resorption is visible.

#### 3.3.4. Plywood-like layer

This layer is approximately 150–250 μm thick, thicker than in the oral plates. Similar to the oral plates, this layer overlies the cancellous layer and consists of plywood-like lamellar layers appositionally added towards the surface (figure 5).

## 4. Discussion

### 4.1. Heterostracan oral plate morphology and growth model

**Figure 6.**
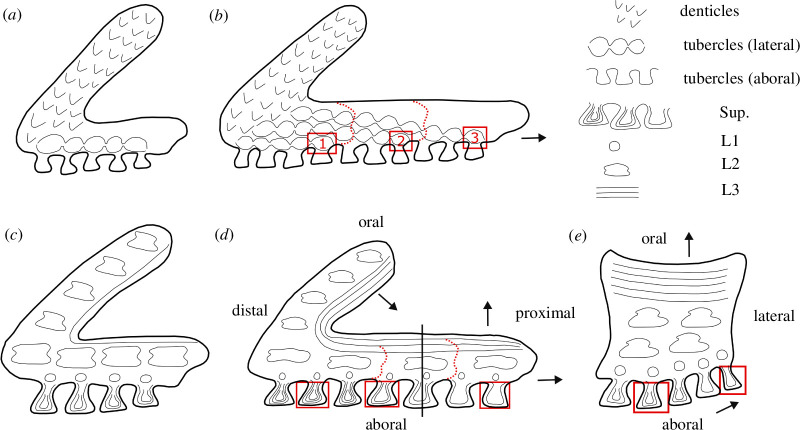
Schematic developmental diagrams of heterostracan oral plates: (*a*,*b*) development of surface morphology in early (*a*) and later (*b*) stages, focusing on the distribution of denticles and tubercles, rectangles indicate lateral tubercle rows (1–3) connecting to aboral tubercle rows; (*c*–*e*) development of internal morphology in early (*c*) and later stages (longitudinal section in *d*, cross-section in *e*), focusing on the internal morphology (superficial layer, L1–L3), rectangles indicate different degrees of tubercle infilling, solid line in *d *indicates location of cross-section in *e*. Dotted lines indicate the extent of the individual growth stages, and arrows indicate directions of growth.

In the lateral oral plate, we observe that on the aboral shaft surface, the tubercles of the more distal rows are more infilled with the centripetally deposited dentine than more proximal tubercles (closer to the articulation of the plate with the ventral shield; see figure 2*j*, right rectangle; see also figure 6*d*, rectangles). Less infilled tubercles demonstrate that the process of dentine deposition is less advanced and that these tubercles are younger, which implies that growth occurs towards the proximal end of the oral plate shaft, with new tubercle addition, along with supporting bone (figure 6*d*, arrows). On the lateral shaft surface, we observe a similar pattern with more aboral rows of tubercles showing a higher degree of infilling with the centripetally deposited dentine than more oral rows (see figure 2*k*, rectangle; see also figure 6*e*, rectangles). This indicates that growth is occurring towards the oral surface (with its less infilled tubercle rows; figure 6*e*, arrows).

The tubercle rows on the lateral surface and the aboral surface are connected. In the median as well as lateral oral plates, we observe that the tubercle ridges on the lateral surface extend and curve over to the aboral side (figure 2*d*–*f*, dotted lines; see also figure 6*b*, rectangles). As new tubercles are added on the aboral surface (as interlocking ridges), a connecting tubercle row is deposited in conjunction on the lateral surface (figure 6*b*, rows 1–3). The tubercles in the corresponding aboral and lateral surfaces show a similar degree of dentine infilling, which supports their coordinated development. We interpret these observations as discrete growth episodes during which the oral plate grows proximally as well as orally (figure 6*d*, arrows). With every proximal growth episode of aboral tubercles, the lateral shaft surface gets successively built from the aboral to the oral with a new row of tubercles (figure 6*d*,*e*).

We also observe successive apposition of growth layers in the plywood-like layer of the oral surface of the shaft and towards the proximal side of the hook (see figure 5*b*,*d*; see also figure 6*d*, arrows). This indicates that the oral plates also grew in depth in addition to length. Both the oral surface of the shaft and the proximal side of the hook are unornamented and show vascular openings (see figure 1*b*–*e* lateral views, rectangles on shaft and oral views, rectangles), which indicates that they must have been covered in soft tissue when the animal was alive. The oral plate hook with its denticles as well as the aboral side of the oral plate with its tubercles were likely the only parts of the oral plate free of soft tissue.

Abrasion of the tubercles around the middle of the aboral surfaces (see figure 1*b*–*d* aboral views; figure 2*j*, left rectangle) reflects repeated abrasive contact with a hard substrate and indicates a locomotion or feeding mode with the animal moving close to the seafloor. The tips of the distal oral plate hooks are also abraded (see figure 2*g*–*i*), which may reflect a mechanical function, e.g. deposit feeding (see [59]).

In one median oral plate specimen, abrasion does not occur on the aboral surface and the tubercles appear pristine (see figure 2*l*, rectangle). This might indicate that the median oral plates did not make contact with a hard substrate. Generally, the median plates do not articulate with the postoral plate (see figure 1*a*), but with the lateral surfaces of the neighbouring oral plate (see [58]). This means the median plates were located in the middle of the apparatus, completely suspended in soft tissue, which might have given them increased protection from making contact with an abrasive substrate. Alternatively, or in addition, the unabraded specimen is smaller in size than the abraded one and might represent an earlier ontogenetic stage during which less contact with hard substrate might have occurred, potentially indicating a change in the mode of locomotion and/or feeding.

### 4.2. Heterostracan dermal skeleton histology

We found that the examined heterostracan dermal skeleton specimens are generally four-layered. The histology we observed corresponds to Keating *et al*. [23], who described the histology of the dermal skeleton of different heterostracan groups. They found that the dermal skeleton comprises the same four layers: the superficial layer of tubercles (Sup.), L1 (compact layer of canals), L2 (cancellous layer) and L3 (plywood-like layer; see figure 3*c* for comparison). Generally, all layers in the examined dermal skeleton specimens are thinner than described by Keating *et al*. [23], but the overall histological architecture is very similar. However, Keating *et al*. [23] observed signs of resorption in layer L1 (compact layer of canals) and also found a coarse fabric of orthogonal Sharpey’s fibres in L3 (plywood-like layer), potentially anchoring the dermal skeleton to the dermis in life, which we did not find in the examined dermal skeleton specimens.

### 4.3. Comparison of the dermal skeleton and oral plate histology

In the oral plates, the superficial layer morphologically manifests as either tubercles on the aboral surface or denticles on the lateral surfaces of the hook. In the dermal skeleton, we only observe tubercles, no denticles. All tubercles and denticles show the same histology, an enameloid cap on top of dentine growing centripetally towards the pulp canals. Only in the dermal skeleton tubercles are the small canals (canaliculi) visible radiating from the pulp canals. However, we also expect these odontoblast processes to be present in the denticles. There are no significant differences in the histology of the oral plate denticles compared to the tubercles on the oral plates and the dermal skeleton. The compact layer of canals (L1) is comparable and has approximately the same thickness in both the oral plate and dermal skeleton specimens examined. Contrary to L1 in the dermal skeleton [23], we observe no evidence of resorption in this layer in our specimens. The layer of open cancellar spaces is thicker in the oral plates than in the dermal skeleton. In the oral plate hook, we observe the cancellar open spaces to be more compact. Vascular spaces cut through the internal structure of the cancellar layer and truncate growth layers (figure 3*e*–*f*; see also electronic supplementary material, S2), suggesting resorption of new vascular spaces, resorption-facilitated expansion of existing vascular spaces or else discontinuous mineralization around existing vasculature. With the exception of the hook region of the oral plates, we do not observe truncation of lamellae in the cancellar layer of the oral or dermal skeleton. The plywood-like layer, which grows in an appositional fashion, is thicker in the dermal skeleton than in the oral plate specimens examined. We do not observe Sharpey’s fibres in the plywood-like layer of the oral plates, which indicates that there might have been some variation in how the oral plates have been connected to the overlying soft tissue.

From these comparisons, we can infer that the histology of the oral plates and the dermal skeleton is generally very similar (with only small modifications) and comprises the same histological layers (superficial layer, compact layer of canals, cancellous layer and plywood-like layer).

### 4.4. Denticle morphogenesis

To test the hypothesis that the denticles on the lateral surfaces of the oral plate hook are homologous to teeth, we analysed their morphogenesis. We previously established development as a necessary criterion for tooth homology; teeth are characterized by patterns of replacement (either via tooth loss or sequential apposition) [8,60,61] and lack of evidence of continuous growth between adjacent odontodes. We therefore looked for these patterns in the denticles by analysing their morphogenesis in order to evaluate if they satisfy the developmental criterion of tooth homology. Together with the topological, developmental and structural criteria, this allows us to establish a hypothesis of primary homology. If the denticles underwent resorption and replacement or apposition, we would expect to see remodelling in the superficial layer as well as in the underlying layer L1. As described above, L2 (the cancellar layer) in the oral plate hook shows truncated lamellae, which could indicate resorption of the vascular canal network. However, we do not observe remodelling in the overlying L1 layer and the superficial layers (with the denticles), which allows us to exclude resorption and replacement. Tooth apposition in the arthrodiran dentition can be recognized by the overlap relationship between the base of the newly added tooth posteriorly and the older, existing tooth (e.g. [10]; figure 2*e*, *g*,*h*). This overlap relationship can be traced along the tooth row, from posterior to anterior, representing timed, patterned addition (replacement without loss) of teeth. A similar apposition mechanism of denticles can be observed in the pharyngeal cavity of thelodonts with the addition of patches and rows of fused denticles showing a strictly polarized sequence and overlapping relationships between the denticles [20]. Even though these polarized denticles occur among otherwise unpolarized denticle aggregates, they can be interpreted as evidence of tooth-like patterns of sequential apposition. In acanthodians, patterned apposition is also present, with rows of teeth being sequentially added along a proximal to distal vector. The teeth reveal clear overlapping relationships and growth arrest lines, with each tooth added onto the distal margin of its predecessor [51]. All these examples of apposition have in common clearly traceable overlapping relationships in the growth structure of the individual odontodes and the absence of continuous growth between them. In the heterostracan oral plates, cracks make it challenging to trace growth layers in the denticles. But all traceable denticles are continuous at their bases, lacking the overlapping relationships observed in arthrodiran, thelodont and acanthodian odontodes and therefore failing the developmental tooth criterion (figure 4; see also electronic supplementary material, S3).

### 4.5. Evaluation of primary homology conjectures for phylogenetic congruence

**Figure 7.**
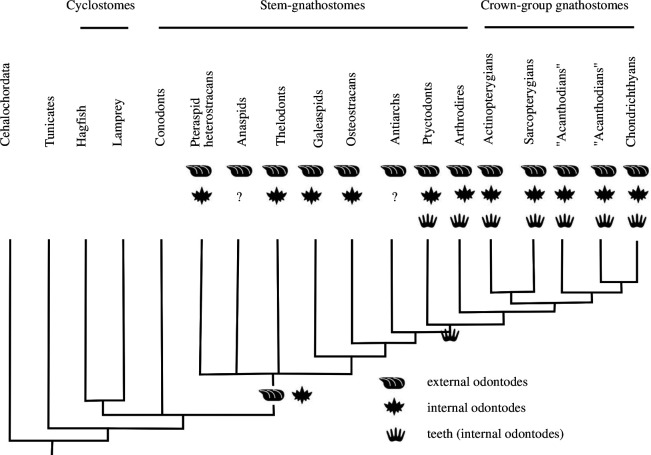
Distribution of external odontodes, internal odontodes and teeth (as a subcategory of internal odontodes), plotted on a phylogenetic tree of vertebrates. Phylogeny adapted from Johanson *et al*. [62]. Question marks denote ambiguous records, and empty tips are not applicable.

To be able to make inferences about the evolutionary history of internal/external odontodes and test for phylogenetic congruence, we analysed the distribution of internal/external odontodes across stem-gnathostomes (figure 7). Various groups of stem-gnathostomes have been shown to possess internal odontodes (figure 7). Conodonts are the only stem-gnathostome group that lacks external odontodes. All other stem-gnathostomes, comprising the so-called ‘ostracoderms’ (heterostracans, anaspids, thelodonts, galeaspids and osteostracans) and the placoderms, share an extensive dermal armour of external odontodes as a conserved feature (figure 7). In addition to external odontodes, pteraspid heterostracans possess internal odontodes on their oral plates in the form of denticles that cover the lateral sides of the hook [27,31] (see also figure 2*a*–c). In the thelodont *Loganellia,* tooth-like structures are also present as whorls associated with denticles [19]. In addition, anteriorly facing denticles have been observed inside the snout of thelodonts. Similarly, in some galeaspids, denticles appear associated with the median dorsal nasal opening (used for respiratory water intake) [63]. Oropharyngeal denticles are also present in osteostracans in association with the median dorsal field [22,64]. Internal odontodes that pass the topological, structural as well as developmental (growth through apposition) tooth criteria occur in placoderms, e.g. the arthrodiran *Compagopiscis croucheri* [10].

### 4.6. Testing the homology conjecture and implications for the evolutionary origin of teeth

We have previously established topology, structure and development as necessary criteria for tooth homology. The denticles of heterostracans are located on the lateral surfaces of the oral plate hooks, which sit inside the oral cavity and develop from oral epithelium. They therefore satisfy the topology criterion to be recognized as teeth. They also possess a typical odontode structure with centripetally growing layers of dentine covered by an enameloid cap satisfying the structural criterion of tooth homology. We additionally analysed development as a criterion for recognizing teeth. Despite their topology and structure, the oral plate denticles do not show signs of apposition or replacement. Instead, their growth is comparable to the continuous growth of the tubercles on the aboral side of the oral plate and the dermal skeleton without any overlapping relationships, but with another morphology. Consequently, heterostracan oral plate denticles do not satisfy the developmental criterion to be recognized as teeth. Heterostracan oral plate denticles appear to be a variety of internal odontodes closely related in terms of their morphogenesis to other internal odontodes on the oral plates (tubercles) as well as external odontodes (dermal skeleton) [8,27,31,58,65–70].

We analysed the distribution of internal/external odontodes to evaluate our primary conjectures of homology for phylogenetic congruence (figure 7). Based on the phylogeny, we conclude that the distribution of external odontodes (dermal skeleton) is a conserved feature with a continuous distribution. The appearance of internal odontodes (like the denticles on the heterostracan oral plates) is rather discontinuous and sporadic among stem-gnathostomes (figure 7). Internal odontodes are not a synapomorphy shared by all stem-gnathostomes and jawed vertebrates; they appear independently in various groups of stem-gnathostomes including heterostracans. Based on this discontinuous distribution, we reject the hypothesis of the heterostracan oral plate denticles being homologous to the teeth of jawed vertebrates.

Our results also help us to inform the discussion around the ‘inside-out’ and ‘outside-in’ hypotheses. The ‘inside-out’ hypothesis postulates the existence of teeth before jaws and their independence from dermal odontodes. Our findings indicate that the denticles on the heterostracan oral plates are not homologous to teeth in jawed vertebrates and therefore do not provide evidence for teeth before jaws. We also found a profound similarity in histology and morphogenesis (no patterns of apposition) of the heterostracan oral and dermal skeleton, which indicates that the oral plate denticles are not developmentally independent of the dermal skeleton but rather share the same developmental origin. The oral plate denticles appear to be a variety of internal odontodes and are closely related to the dermal skeleton. Our results are more compatible with the ‘outside-in’ hypothesis, which proposes the evolutionary origin of teeth from the dermal skeleton based on the spread of odontogenic competence from extra-oral to oral epithelia. This is also supported by the fact that in heterostracans the denticles are located near the mouth opening, on the lateral surfaces of the oral plate hook, in very close association with the external odontodes. Odontogenic competence spread from dermal epithelium to oral and pharyngeal epithelia independently several times in heterostracans, thelodonts, galeaspids and osteostracans, evidenced by the phylogenetic distribution of internal odontodes (denticles) in the oral, pharyngeal or nasal region of these groups (see figure 7). The internal odontodes of stem-gnathostomes are not directly homologous to gnathostome teeth. But their capacity to form internal odontodes through the spread of odontogenic competence from extra-oral to oral epithelium might have been exapted to a tooth function in association with the origin of jaws. Our findings support this scenario and provide additional evidence for the ‘outside-in’ hypothesis.

## 5. Conclusion

The evolutionary relationship of teeth in jawed vertebrates to tooth-like structures in jawless vertebrates is poorly understood. Studying tooth-like structures in heterostracans, a group of extinct early jawless vertebrates, allows us to develop hypotheses regarding their homology with teeth, to better understand this evolutionary transition and shed light on the debate around the ‘inside-out’ versus ‘outside-in’ theory of tooth evolution. Using high-resolution synchrotron scans, we investigate the histology and morphogenesis of heterostracan oral plates to compare to the dermal external skeleton. Based on our observations, we devised a general model of growth for heterostracan oral plates and observed similarities in the histology of the oral plates and the heterostracan dermal skeleton. The analysis of the oral plate denticles does not yield evidence for patterns of replacement or apposition. We observe denticle growth layers to be continuous, rather than showing overlapping bases associated with appositional tooth addition. Growth is instead comparable to the tubercles on the aboral surface of the oral plate and the dermal skeleton. Evaluating the phylogenetic distribution of internal odontodes in comparison to external odontodes reveals the discontinuity of this trait across phylogeny. Internal odontodes are not a synapomorphy shared by stem-gnathostomes and jawed vertebrates. Therefore, we reject the tooth homology hypothesis for heterostracan oral plate denticles. The capacity to form internal odontodes evolved independently many times in various groups of stem-gnathostomes and might have been co-opted to a tooth function in gnathostomes later in evolutionary history. Our findings contradict the ‘inside-out’ hypothesis of tooth evolution, which proposes the origin of teeth before jaws and their independent development from the dermal skeleton. Our results rather support the ‘outside-in’ theory, which argues for an evolutionary origin of teeth closely associated with the dermal skeleton; odontogenic competence spreading from extra-oral to oral epithelia and forming the denticles on the oral plates.

## Data Availability

Data are available at the University of Bristol data repository, data.bris, at https://doi.org/10.5523/bris.r8f8wgpe3ez52eeebnh96jofv. Supplementary material is available online [71].
